# Smart nanoparticles and microbeads for interventional embolization therapy of liver cancer: state of the art

**DOI:** 10.1186/s12951-023-01804-7

**Published:** 2023-02-06

**Authors:** Sitong Wu, Kevin Fan, Qi Yang, Zhao Chen, Yi Hou, Yinghua Zou, Weibo Cai, Lei Kang

**Affiliations:** 1 Department of Nuclear Medicine, Peking University First Hospital, Beijing, 100034 China; 2 Department of Interventional Radiology and Vascular Surgery, Peking University First Hospital, Beijing, 100034 China; 3 Departments of Radiology and Medical Physics, University of Wisconsin, Madison, WI 53705 USA; 4 College of Life Science and Technology, Beijing University of Chemical Technology, Beijing, 100029 China

**Keywords:** Microspheres, Nanoparticles, Transarterial chemoembolization, Multifunctional, Degradable, Hepatocellular carcinoma

## Abstract

The process of transcatheter arterial chemoembolization is characterized by the ability to accurately deliver chemotherapy drugs with minimal systemic side effects and has become the standard treatment for unresectable intermediate hepatocellular carcinoma (HCC). However, this treatment option still has much room for improvement, one of which may be the introduction of nanomaterials, which exhibit unique functions and can be applied to in vivo tumor imaging and therapy. Several biodegradable and multifunctional nanomaterials and nanobeads have recently been developed and applied in the locoregional treatment of hepatocellular cancer. This review explores recent developments and findings in relation to micro-nano medicines in transarterial therapy for HCC, emerging strategies to improve the efficacy of delivering nano-based medicines, and expounding prospects for clinical applications of nanomaterials.

## Introduction

Primary liver cancer is the third most common cause of cancer-related mortality worldwide, with an estimated incidence of  >  1 million cases by 2025 [1]. Furthermore, HCC accounts for > 80% of primary liver cancers worldwide, with incidence rates of HCC in the USA having increased two to three times in the past three decades [1, 2]. Primary liver cancer is also the fourth most common malignant tumor in China [3], indicating the severity this cancer has on the life and health of the Chinese people. Currently, the main clinical treatment methods for liver cancer include surgical resection, liver transplantation, transcatheter arterial chemoembolization (TACE), ablation, and systemic treatment (targeted therapy, immunotherapy, et al.) [4]. It is important to recognize that 75% of the blood supply of a healthy liver originates from the portal vein, while the blood supply in an individual with primary liver cancer originates from the hepatic artery [5], compared with systemic chemotherapy, transarterial treatment has the prominent advantage of accurately delivering chemotherapy drugs and minimizing systemic side effects. Therefore, TACE has become the standard treatment for unresectable intermediate HCC and accurately treats cancer lesions with high safety [6].

Conventional transarterial chemoembolization (cTACE) is the process of mixing chemotherapeutic drugs and ethiodized oil into an emulsion for embolization. However, the ethiodized oil is liquid and its particle size is not fixed, meaning it often has adverse reactions during application which can include embolization to normal liver tissues, entrance into non-target organs, with the ethiodized oil also obscuring arterially-enhancing tumors and limiting the detection of residual tumors. Therefore, drug-eluting bead TACE (DEB-TACE) was developed to overcome these defects. Compared with cTACE, DEB-TACE allows for sustained and stable drug release [7], causing the local tumor to reach a higher blood concentration and a significant reduction in drug-related toxicity and hepatotoxicity [8].

**Fig. 1:**
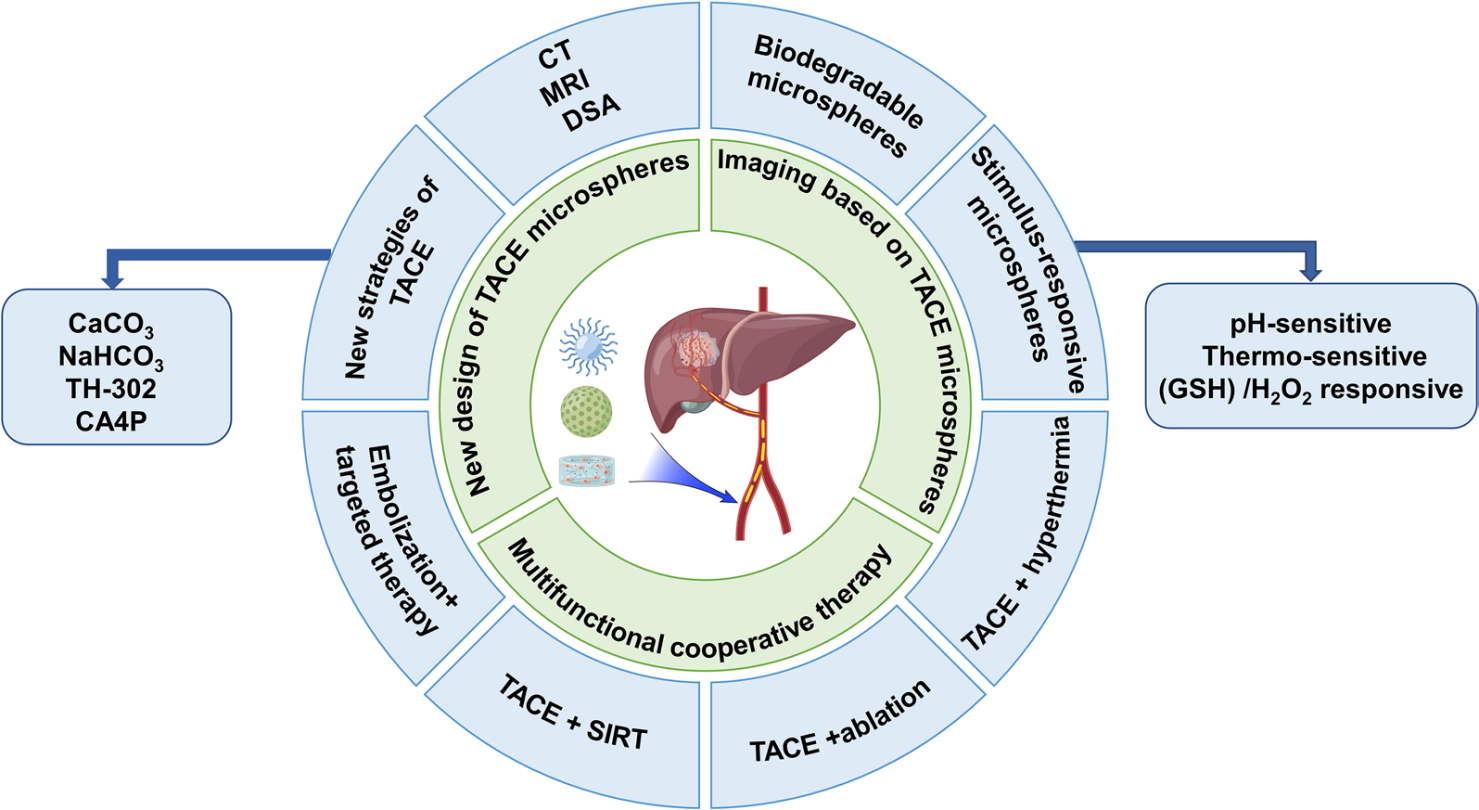
Smart nanoparticles and microbeads for tumor interventional embolization therapy

However, TACE still has much room for improvement, and many smart nanomaterials with functions of detection, degradation, and synergistic therapy have been developed in recent years (Fig. 1). Herein, this review introduced the recent developments and findings of micro-nano medicines in transarterial therapy for HCC, the emerging strategies to improve the efficacy of delivering nano-based medicines and expound prospects for clinical applications of nanomaterials.

## Current technology and design of TACE microspheres

### Biodegradable microspheres

Despite cTACE being recommended as the first-line palliative treatment option for patients with unresectable HCC, the use of ethiodized oil mixed with chemotherapeutic drugs can result in a higher incidence of systemic, adverse effects on tumors [9]. Drug-eluting bead TACE (DEB-TACE) provides an attractive alternative regimen, with experimental and clinical studies indicating that DEB-TACE is associated with a better objective response, disease control, and lower complication risk compared to cTACE treatment [10–12].

### Benefits of degradable nanomaterials

Currently, the common commercially available microspheres on the market are mainly composed of non-biodegradable polyvinyl alcohol (PVA) and polyethylene glycol (PEG) [13]. However, these non-biodegradable microspheres have some limitations: (1) the permanent embolism in non-target arteries causes liver tissue infarction or gallbladder infarction [14]; (2) non-biodegradable microspheres cause permanent obstruction, which would inhibit effective retreatment following the recurrence of HCC [15]. Compared to non-biodegradable microspheres, the advantages of degradable microspheres include a potential reduction in the occurrence of the post-embolization syndrome, avoidance of ischemia-induced neoangiogenesis [16], and the temporary protection of normal liver parenchyma during TACE [17].

### Major classes of degradable nanomaterials

At present, degradable microspheres have become the focus of embolic agent development, with common microspheres including chitosan-cellulose microspheres, polyethylene glycol methacrylate (PEGMA) microspheres, poly(D,L,-lactic acid) (PDLA, PLLA) and poly(lactic-co-glycolic acid) (PLGA) microspheres[18, 19].

When looking at chitosan-cellulose microspheres, chitin and chitosan can be harvested from the shells of crustaceans and are degraded by lysozymes that are present in the human body. They can be used as carriers for chemoembolization due to their biocompatibility, biodegradability, and nontoxic characteristics [20]. Chitosan magnetic microparticles (CMM) are a special class of chitosan microparticles that have been developed and widely applied in the delivery of anticancer drugs [21]. About liver cancer, CMM would enter via the hepatic artery, and stops at the targeted tissue through the application of an external magnetic field. When further looking at chitosan nanoparticles, Li et al. [22] indicated that the encapsulation of carboplatin into chitosan magnetic microparticles, or carboplatin-Fe@C-loaded chitosan nanoparticles, have a dual role–drug carrier and hyperthermia.

Poly(lactic-co-glycolic acid), or PLGA, is a hydrophobic and degradable polymer. It undergoes hydrolysis in the body to produce lactic acid, and glycolic acid [23], which are biodegradable metabolite monomers and easily metabolized by the body. There is very minimal systematic toxicity when using PLGA [24], and the improved antitumor efficacy of drug-loaded PLGA microspheres in transarterial chemotherapy has also been well documented [25].

### Stimuli-responsive nanomaterials

Ethiodized oil and DEB can cause catheter blockage due to their adhesiveness, obstructing transcatheter delivery of the drug and subsequent treatment. In recent years, some new intelligent response embolic agents have been developed to counteract these shortcomings. These include thermo-sensitive hydrogels, which are free-flowing liquids with a lower critical solution and can be transformed into a gel at physiological temperatures in vivo [26]. Additionally, stimulus response nanoparticles can respond to the high acidity [27], and the presence of reactive oxygen species and reducing substances that characterize the tumor microenvironment (TME) undergo a transformation of phase state or aggregation state to achieve vascular resistance [28].

### Redox-responsive

**Fig. 2:**
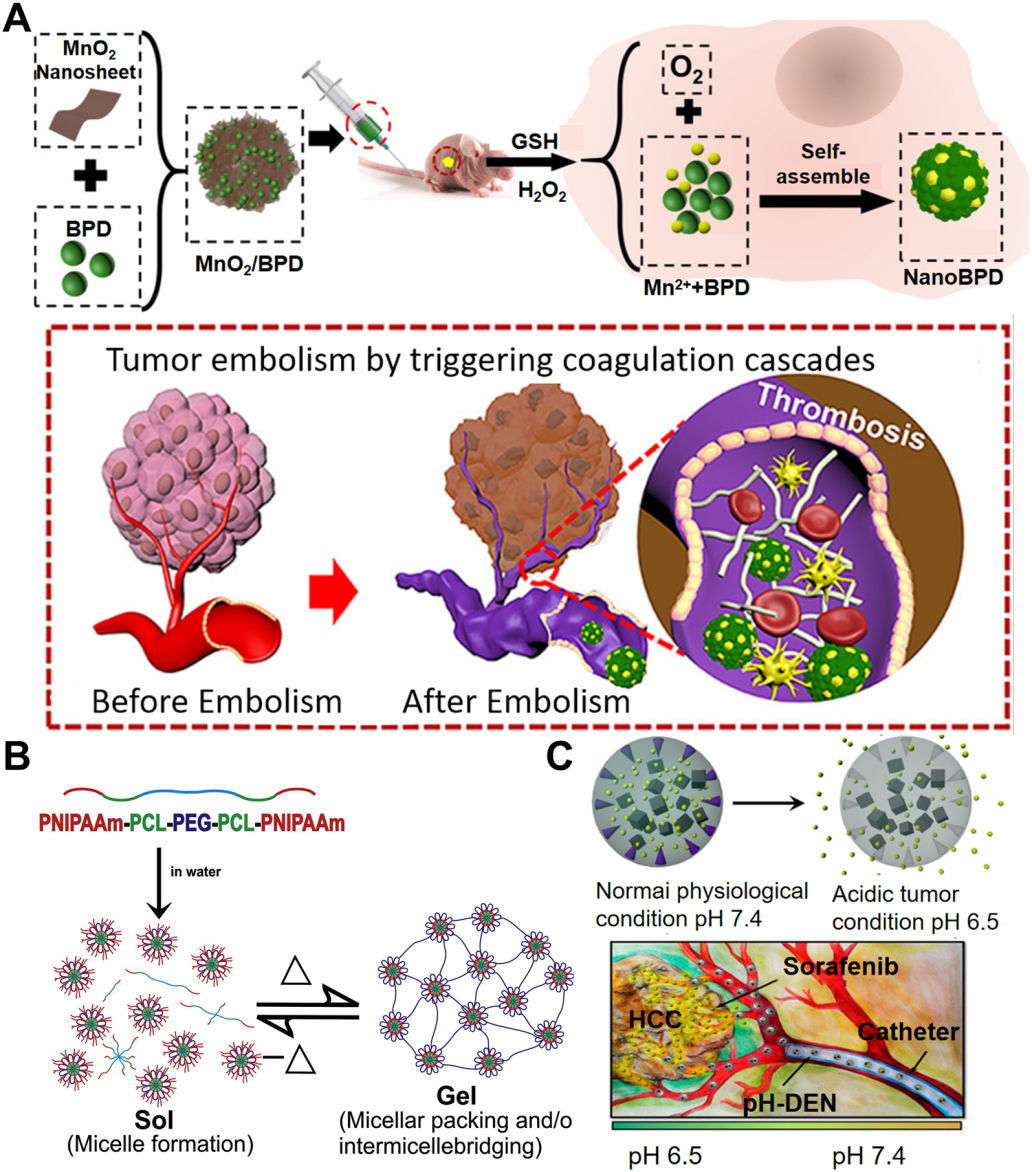
Stimulus-responsive nanoparticles for tumor embolization therapy. **A** The MnO_2_/BPD fabrication and thrombosis formation induced by Interventional PDT. Reproduced with permission from Ref. [32]. Copyright 2020, American Chemical Society. **B** The fabrication and mechanism of the thermo-sensitive hydrogel. Reproduced with permission from Ref. [35]. Copyright 2014, Elsevier Ltd. **C** The mechanism of pH-sensitive nanoparticles for tumor embolization therapy. Reproduced with permission from Ref. [35]. Copyright 2016, American Chemical Society

In O_2_-dependent therapies, such as photodynamic therapy (PDT), H_2_O_2_ has been used as the substrate of O_2_ generation to increase therapeutic effect [29]. At the same time, antioxidant glutathione (GSH) also has a high concentration in tumors [30] and plays an important role in the increased resistance of tumorigenic cells to treatment [31]. Therefore, it is of great significance to design a nano platform that combines GSH consumption with ROS generation. One example is MnO_2_ nanosheets designed by Wang et al. that was used as the carrier for a benzoporphyrin derivative (BPD) (MnO_2_/BPD) [32]. Under the conditions of high levels of GSH and H_2_O_2,_ the nanosheets were quickly reduced to large amounts of Mn^2+^ and BPD, resulting in the generation of ROS and the depletion of GSH which inhibited tumor growth and improved the therapeutic efficacy of PDT (Fig. 2A). The enhanced effect of PDT could be able to kill tumor vascular endothelial cells and amplify the effect of the tumor embolization effect by inducing the coagulation cascade.

### Thermo-sensitive

Thermo-sensitive hydrogels are three-dimensional hydrophilic polymer networks that contain considerable water content and are well-regarded as nontoxic and biocompatible polymers [33]. They have been widely studied for potential applications in biomedicine and drug delivery biomaterial [34].

Among many materials, poly(N-isopropylacrylamide) (PNIPAAm) has been widely used. PNIPAAm exhibits a reversible phase transition in response to a lower critical solution temperature (LCST) of 32 °C (Fig. 2B) [34, 35]. Huang et al. [36] investigated thermos-sensitive liquid embolic hydrogels containing poloxamer 407 as liquid embolic agents for TACE therapy relating to liver cancer. These composite hydrogels were injectable at ambient temperature (< 25 °C) and transformed into a gel at body temperature (37 °C) [37]. The composite hydrogels demonstrated successful occlusion within the VX2 liver cancer model. However, PNIPAAm can be cytotoxic due to the presence of unreacted monomers or toxic moieties arising under the hydrolysis of chains [38]. Alternative temperature-sensitive gels with biocompatibility have been designed to avoid this shortcoming: poly(N-vinylcaprolactam) (PVCL), poly(vinyl methyl ether) (PVME), poly(N, N-dimethylaminoethyl methacrylate) (PDMAEMA), etc. [39].

### pH-sensitivity

The extracellular region of liver tumors is acidic due to excessive glycolysis and poor perfusion [40]. Therefore, pH-sensitive injectable hydrogels have recently emerged as a promising approach for selective drug delivery to tumor tissues. For hydrogels with specific acidic and/or basic pendant groups, water uptake by such polymers mainly takes place due to the pH of the external solution. Moreover, given the presence of specific charged substances in the structure of the swelling network, pH-sensitive polymer hydrogels will release drugs based on the specific pH of the external solution [34]. When looking at the applications of PCL-PEG-SM, a copolymer comprised of poly(ε-caprolactone), sulfamethazine, and poly (ethylene glycol), it was loaded with doxorubicin (DOX) and underwent a sol-to-gel phase transition through the variation of environmental pH to create a gel region that embodied the physiological conditions (pH 7.4) and low pH conditions at a tumor site (pH 6.5–7.0) [41]. This hydrogel successfully embolized a hepatic tumor of a VX2 rabbit tumor model while maintaining sustained release of DOX. Furthermore, an acidic pH-triggered drug-eluting nanocomposite containing a pH-responsive additive (pH-ADT) has been developed for TACE (Fig. 2C). pH-ADT can be formed as a stable solid state at a neutral pH. An acidic environment (< pH 7.4) destabilizes the pH-ADT because of the ionization of imidazole functional groups, which causes a phase transition of the pH-ADT into a charged water-soluble form [42]. It is demonstrated that the drug release could be triggered by a response to the acidic microenvironment caused by embolization-induced hypoxia.

## Multifunctional nanoparticles for synergistic cancer therapy

There are still some limitations of TACE, such as incomplete embolization of the supply arteries of the tumor, which may lead to tumor progression and recurrence [43]. Embolization, combined with multiple therapies, such as hyperthermia, radiation, and ablation are potential ways to enhance the therapeutic effect of liver cancer.

### TACE in combination with thermal ablation

Ablation of liver cancer is a form of treatment that utilizes the guidance of medical imaging technology to target the tumor foci and locally kill the tumor tissue directly through physical or chemical methods [44]. Ablation therapy is mainly applicable to patients with BCLC stage A who are not candidates for surgical intervention [45]. Forms of treatment include radiofrequency ablation, microwave ablation, absolute ethanol injection therapy, cryoablation, high-intensity focused ultrasound (HIFU), laser ablation, irreversible electroporation, etc. [46]. HIFU is a non-invasive operation that focuses high-intensity ultrasound energy to ablate and destroy the tumor tissue and does no damage to normal tissues in the acoustic-propagating path [47]. However, the efficiency of HIFU for deep-seated liver cancer is low. The delivery of an ablation sensitizer to the tumor site through the hepatic artery can increase the effect of ablation on tumor cells.

**Fig. 3:**
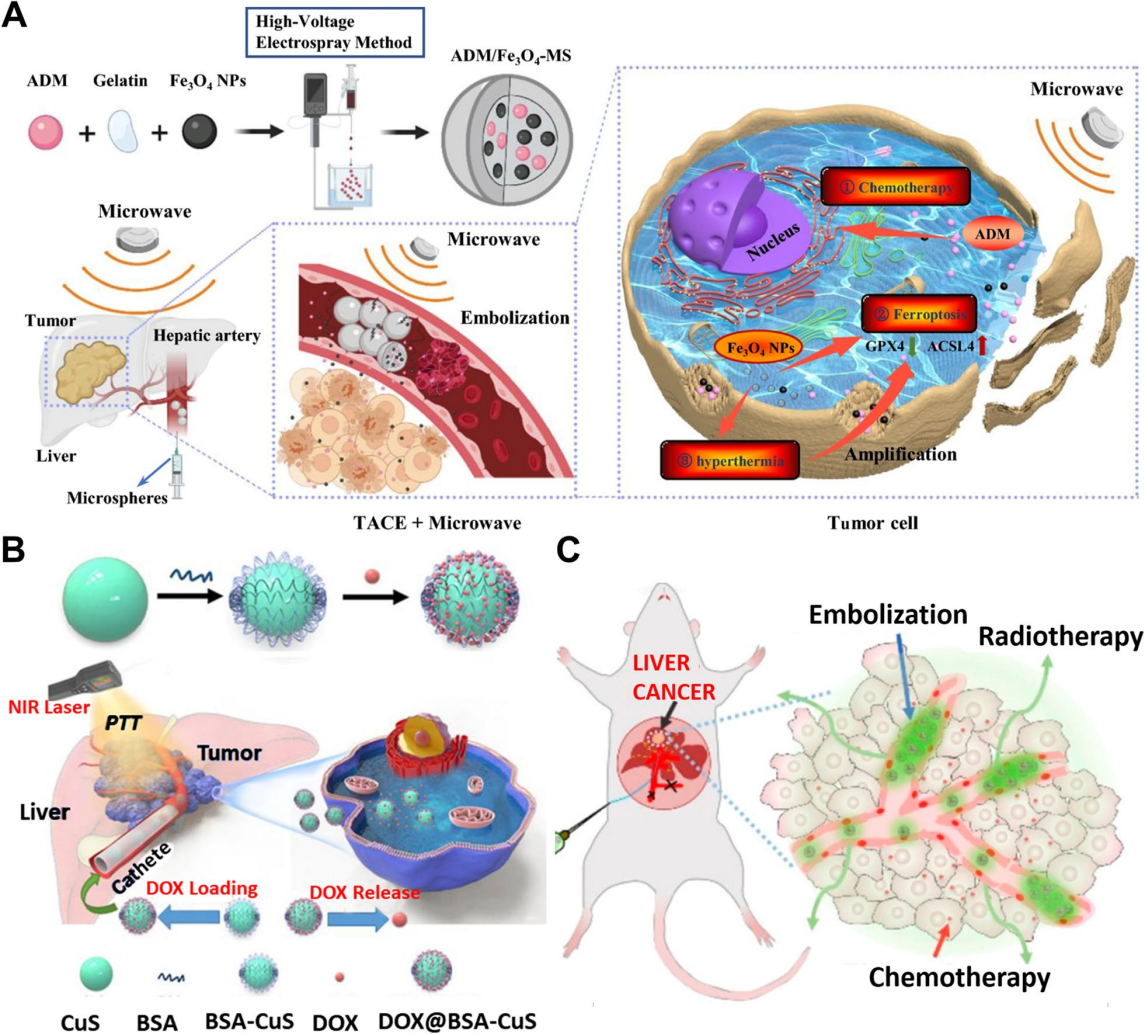
Synergic therapeutic nanoparticles for tumor embolization therapy. **A** The preparation process of Fe_3_O_4_ nanoparticles and its TACE treatment mode. Reproduced with permission from Ref. [49]. Copyright 2020, Springer Nature. **B** Schematic illustration of DOX-encapsulated and near-infrared (NIR)-responsible nanoparticles preparation and chemo-photothermal treatment of orthotopic liver cancer. Reproduced with permission from Ref. [55]. Copyright 2021, Elsevier Ltd. **C** Schematic illustration of ^131^I microbeads for radio-chemoembolization. Reproduced with permission from Ref. [63]. Copyright 2021, American Chemical Society

Therefore, Fe_3_O_4_-integrated PLGA capsules were developed as multifunctional nanosystems and ultrasound synergistic agents for transarterial chemoembolization, diagnostic imaging, and enhanced HIFU (Fig. 3A) [48]. Superparamagnetic iron oxide nanoparticles (SPIONs), which function as microwave-sensitive material, absorb microwave energy through unpaired electrons (Fe^2+^, Fe^3+^). When these excited electrons return to the ground state, they release phonons that allow for the conversion of microwave energy into heat energy [49]. In a microwave field, SPIONs in microspheres can rapidly increase a high temperature to kill tumors and further induce ferroptosis of tumor cells.

### TACE in combination with hyperthermia

The use of local hyperthermia to induce the death of cancer cells can enhance the effect of chemotherapy. Local hyperthermia includes arterial embolization hyperthermia (AEH). Treatment at temperatures between 40 and 44 °C is cytotoxic for cells in an environment with a low pH and low oxygen partial pressure, due to insufficient blood perfusion within tumor tissue caused by embolization [50]. Moreover, the effects of chemotherapy drugs are enhanced at an increased temperature, and it has been shown for DOX that the addition of hyperthermia to chemotherapy can counteract drug resistance [51].

### Magnetic hyperthermia

Magnetic hyperthermia is cancer-targeted hyperthermia that is implemented through the use of basic principles of TACE in conjunction with an external magnetic field to localize magnetic material to a tumor site, which results in increased temperatures at the site of the magnetic material; normal tissues do not generate heat due to the absence of ferromagnetic materials. Recently, magnetic microparticles have been investigated for embolization and hyperthermia. It is demonstrated that treatment with PLGA microsphere containing Fe_3_O_4_ particles causes tumor necrosis and suppresses associated tumor angiogenesis in VX2 liver tumors [52]. More importantly, the phase transformation of PLGA from the glassy state to the rubbery state, which is induced by magnetic heating effects enhanced microparticle aggregation, subsequently reducing the impact on liver function. Zhao et al. [53] investigated a magnetic drug carrier system that contained micron-sized iron powder, barium ferrite (BaFe_12_O_19_), and carbon-coated iron nanocrystals (CCINs). BaFe_12_O_19_ is not magnetized before the nanoparticles are injected into the hepatic artery. However, BaFe_12_O_19_ has strong magnetic properties when magnetized under the action of an external magnetic field, which attracted iron powder and CCINs to form large particles in an external static magnetic field after they were infused into the liver tumor. As a result, the magnetic nanoparticles thoroughly embolized the supplying artery of the tumor. The magnetic induction temperature could reach a maximum level in one minute to kill residual cancer cells in an external alternating magnetic field. More importantly, the powder particles adhered to adjacent tissues after AEH, allowing the use of these clumps for repeated hyperthermia. Pure iron is 2.6 times the magnetic intensity of saturated magnetic. Therefore, Li et al. [22] designed magnetic microparticles based on pure iron which possesses higher saturation magnetization, and the tumor temperature may be controlled by adjusting nanoparticle concentration and magnetic field current strength.

### Photothermal therapy (PTT)

Photothermal nanoparticles can be used for the PTT of tumor cells by converting absorbed light energy into heat energy under near-infrared light [54]. Gold nanomaterials, CuS nanocrystals (Fig. 3B), polydopamine, carbon nanotubes, and graphene have all been reported as the main PTT agents [55, 56]. Specifically, polymers such as polydopamine, with upper critical solution temperature (UCST) properties are perfectly suitable for the construction of drug delivery platforms to achieve a controllable release pattern [56]. The solubility and drug release rate of photothermal microspheres increase substantially when a specific temperature threshold is achieved, which facilitates hyperthermia and chemotherapy to function simultaneously. The co-delivery of photothermal nanoparticles and chemoembolization drugs through hepatic arteries can be used to mediate simultaneous PTT and chemoembolization therapy of cancer cells. In addition, Hollow Gold Nanospheres (HAuNS) are a novel class of gold nanoparticles capable of converting absorbed photographic energy into heat [57]. Near-infrared laser irradiation both elevates the temperature of the treated tissue and destroys the nanospheres, promoting the release of the cytotoxic drugs payload directly into the tumor [58, 59]. However, the cost of noble metals such as gold is high, therefore MoS_2,_ a non-noble metal-based PTT agent serves as an alternative with lower cost, stronger light absorption in near-infrared regions, higher photothermal conversion efficiency, and better biocompatibility [60].

### TACE in combination with selective internal radiation therapy (SIRT)

SIRT is a catheter-based procedure that delivers internal radiation to liver tumors via the hepatic arterial vasculature in the form of microspheres or ethiodized oil. Among the more promising of these radiotherapeuticals are microspheres. Only three types of microspheres are commercially available: Yttrium-90 (^90^Y)-resin microspheres (Sir-Spheres^®^), ^90^Y-glass microspheres (Therasphere^®^), and holmium-166 (^166^Ho)-PLLA microspheres (QuiremSpheres^®^) [61]. As well as these microbeads, other radionuclide labeled nano-based or polymeric microspheres are currently being developed. An overview is given in Table 1.

**Table 1:** Radiolabeled microspheres in clinics nowadays

Radionuclides	Microbeads materials	Trade name	Refs.
^90^Y	Glass	TheraSphere^®^	[62]
^90^Y	Resin	SIR-Spheres^®^	[62]
^131^I	Tyrosine to PVA	–	[63]
^188^Re	Human serum albumin (HSA)	–	[64]
^188^Re	Lipid nanocapsules (LNC)	–	[65]
^166^Ho	PLLA	Quiremspheres^®^	[66]

### ^*90*^*Y and *^*166*^*Ho*

^90^Y is a pure beta-emitter with a half-life of 64 h and a maximum energy of 2.27 MeV [67]. Since ^90^Y released beta energy has a maximum penetration range of 11 mm within soft tissue [68], embolization microspheres containing ^90^Y are injected intra-arterially to kill tumor cells. However, the two kinds of 90Y-microspheres on the market have their disadvantages. The high density of glass microspheres increases the chance of premature intravascular settling and falling back into the gastroduodenal thus giving rise to undesirable side effects [61]. Though the density of resin microspheres is low, the activity of resin microspheres is 50 Bq per sphere, which means a greater number of administered spheres is required to deliver the same radiation dose [69], resulting in a greater likelihood of non-target embolization and particle reflux into unintended vasculature.

Besides, it is important to recognize that preprocedural hepatic arterial mapping is standard and necessary before ^90^Y radioembolization to maximize efficacy and reduce potential nontarget embolization. In this process, Technetium-99 m macro aggregated albumin (^99m^Tc-MAA) is injected in the arterial territory of the targeted tumors to evaluate lung shunt fraction by nuclear imaging with single-photon emission computed tomography (SPECT) as well as to avoid the inadvertent deposition of microspheres in organs other than the liver [70]. However, the size of ^99m^Tc-MAA particles is slightly smaller than that of commercially available ^90^Y microspheres, which may induce an overestimation of the lung shunt with ^99m^Tc-MAA [69]. Therefore new imageable microbeads with proper density, and high specific activity are the focus of research and development, and nano microspheres are the most ideal material. For instance, the PLLA microbeads are not only biodegradable but also have a density comparable to blood [69]. More importantly, ^166^Ho provides an alternative to ^90^Y and possesses potentially superior imaging characteristics, specifically in relation to its paramagnetic properties which potentially allow for using MRI, and better visibility by SPECT due to its gamma-ray emission (81 keV, 62%) [71]. So that ^166^Ho-PLLA microspheres can be located and quantitative on MRI, and the safety and effectiveness of microspheres have been verified in several clinical trials [72–74]. Some ongoing clinical research will further provide new knowledge relating to dosimetry and personalized patient treatment [75].

### *Iodine-131 (*^*131*^*I)*

^131^I-labeled ethiodized oil has also been used for SIRT therapy of HCC. ^131^I emits both β and γ rays with a half-life of 8 days, and its β particles have a maximum energy of 0.6 MeV and a maximum path length in the tissue of 2.3 mm [76]. Radioactive lipiodol accumulates in cancer tissues and decreases irradiation damage to non-cancer tissues due to its selectively deposits in nodules of hepatocellular carcinoma. However, in a representative clinical study of 15 patients, the poor tumor retention of ^131^I-ethiodized oil results in ectopic embolization in the lungs, which causes adverse effects such as interstitial pneumonia [77]. Therefore, slowly degrading or nondegradable multifunctional embolic agents with stable ^131^I-labeling were developed to increase tumor-specificity and produce a synergetic antitumor effect at a low dose (Fig. 3C) [63].

### *Rhenium-188 (*^*188*^*Re)*

^188^Re decays with a half-life of 17 h to stable ^188^Os by emission of β particles with maximum energies of 2.12 MeV [78]. ^188^Re with a shorter half-life and similar energy of β rays compared to ^90^Y, is a promising radionuclide proposed for TARE of hepatic tumors. It also emits 155 keV gamma emission when being used for imaging [79]. In addition, ^188^Re can be obtained from^188^W/^188^Re generators [76], which is convenient for research and routine clinical use. Human serum albumin (HSA) microsphere can serve as an ideal radionuclide carrier for ^188^Re in liver cancer treatment due to its high mechanical stability and chemical stability to resist hydrolysis and radiolysis [64]. Lipid nanocapsules (LNC), a nano vector with biomimetic properties, are also a potential ^188^Re nanocarrier as it modifies the biodistribution of entrapped therapeutic agents [65, 80].

Additionally, because the half-life of ^188^Re is as short as 16.9 h, it cannot be transported and stored for a long time. Furthermore, the injection activity of ^188^Re must also be around 2.8 times higher than that of ^90^Y to reach an equivalent treatment effect, which exposes physicians and patients to more radiation [81]. Currently, preliminary results of the phase 1 ^188^Re-lipiodol I clinical trial have clearly shown that ^188^Re-lipiodol displays remarkable biodistribution characteristics and tumor targeting, as well as the highest in-vivo stability among all radiolabeled Lipiodol compounds reported to date [82].

### Embolization in combination with targeted drugs

Molecular targeting drugs, or tyrosine kinase inhibitors (TKIs) such as lenvatinib, sorafenib, and so on are the first line of treatment in systematic therapy for liver cancer [83, 84]. However, systemic exposure to TKIs following oral intake can lead to severe side effects due to a lack of specificity [85]. In addition, the ischemia and hypoxic conditions induced by transarterial embolization also contribute to angiogenesis in the periphery of the tumor. Furthermore, studies have indicated that an increase in serum vascular endothelial growth factor (VEGF) and serum hypoxia-inducible factor 1 alpha (HIF-1alpha) levels after transarterial embolization have been correlated to poorer patient outcomes [86]. Therefore, embolization combined with the local administration of antiangiogenic agents is a new therapeutic strategy. Sorafenib is a potent multikinase inhibitor that inhibits angiogenesis by utilizing the targeted blockage of VEGF and platelet-derived growth factor receptors while also inhibiting cell proliferation [87]. Compared with traditional TACE, the treatment with embolic microspheres loaded with sorafenib exhibits remarkable anticancer therapeutic effects, with low expression of CD34, an angiogenic marker, in tumor tissues [88]. Similar to sorafenib, two other antiangiogenic agents of clinical interest, sunitinib and vandetanib, can be efficiently loaded into beads [89, 90].

## New strategies for TACE

The hypoxia and low acid environment of tumors caused by embolization may lead to tumor recurrence and progression. Accordingly, it is very important to design particulate systems that can combat post-embolization hypoxia and prevent neoangiogenesis to maximize the therapeutic responses of TACE in HCC.

TACE, combined with the optimization of the tumor microenvironment is a new strategy for enhancing the treatment of HCC. Zhang et al. [91] presented CaCO_3_-containing embolic agents to improve cancer treatment via tumor pH neutralization because CaCO_3_ is pH-sensitive. Drug-loaded CaCO_3_ particles can not only release drugs in response to low pH but also cause a significant increase in intratumor pH and modulate the tumor immune microenvironment. Moreover, the overall morphology of microspheres after the decomposition of CaCO_3_ is not affected.

Drug-eluting embolic systems containing NaHCO_3_ were designed for the rapid release of drugs by CO_2_ gas generation in the slightly acidic TME while maintaining the particle structure for occlusion of tumor-feeding vessels [92, 93]. It is expected that the developed TME-responsive gas-generating microspheres can be efficiently applied to TACE.

Low oxygen levels are rarely observed in normal tissues, but hypoxic regions are common in tumors and contribute to chemotherapy resistance [94]. The interruption of blood supply during the TACE process also results in severe hypoxia of the tumor. Therefore, hypoxia-activated prodrugs (HAP) such as TH-302 can be used for TME-targeted cancer therapy. TH-302 prodrugs can be rapidly converted into active components under hypoxic conditions of the TME to kill tumor cells [95]. PLGA microspheres have also been designed to deliver TH-302 and exert physical embolism of the blood supply of tumor tissue [96].

In addition, because most embolic microspheres passively target tumors due to the enhanced permeability and retention (EPR) effect, the dose accumulated in other tumor sites is limited [97]. The addition of tumor vascular-specific destructors such as combretastatin A-4 phosphate disodium (CA4P) can increase the permeability of blood vessels and cell membranes, maximizing the accumulation of chemotherapy drugs in the tumor site, and further reducing toxic and side effects [58]. Additionally, because CA4P specifically blocks and destroys the blood vessels supplying tumors, it can inhibit the formation of new blood vessels.

## Visualization of TACE agents

When looking at DEB-TACE visualization, the current method of visualization involves mixing the contrast media with microbead suspension, and it is difficult to observe the precise location of the embolization microsphere on computed tomography (CT) or magnetic resonance imaging (MRI).

The visualization of embolic microspheres and nanomaterials can provide real-time intraprocedural feedback and may remain detectable in follow-up imaging. Some new embolic microspheres, that are detectable by the addition of MRI contrast agents, have been recently introduced. SPIONs and gadolinium chelates are common contrast agents which are co-encapsulated for the MRI of microsphere delivery [98–102]. SPIONs are magnetic nanoparticles that have various characteristics such as magnetic targeting, biocompatibility, and degradability [103]. SPIONs can not only permit MRI detection given strong T2 and T2* relaxation effects but also allow for effective drug release [99]. PLG sorafenib iron oxide microspheres were developed for quantitative MRI, and the concentration of microspheres can be estimated by evaluating changes in tumor T2* values MRI [98]. In addition, microspheres containing drug-loaded temperature-sensitive liposomes, holmium ions (T2* contrast agent) and Gd (HPDO3A) (H_2_O)] (T1 contrast agent) to accomplish triggered drug release have also been reported [101].

It is still a challenge to evaluate the treatment effect after c-TACE via CT because of the high density of ethiodized oil. PLGA microbeads and 2,3,5-triiodobenzoic acid (TIBA), a contrast agent for CT imaging, have been developed for locoregional delivery of sorafenib and lesion monitoring on follow-up CT imaging [104]. However, the synthesis of such copolymers is complicated, and there is a need for a universal method to economically and efficiently produce radiopaque DEBs. Tantalum nanoparticles (Ta NPs) have the potential to be an excellent non-iodinated contrast media due to their biocompatibility and excellent X-ray attenuation ability [105]. A one-step electrospray method was used to prepare intrinsic radiopaque calcium alginate microspheres loaded with tantalum nanoparticles [106]. These nanoparticles provided the real-time location of the embolic microspheres and exhibit excellent X-ray-visibility properties for up to 4 weeks.

3D vascular reconstruction can be carried out with the help of CT post-processing technology. MRI is the preferred imaging technology for evaluating the therapeutic efficacy of liver cancer. Together, both modalities offer significant individual and specific diagnosis data to physicians. Therefore, to obtain dual-modality imaging, an innovative strategy has been proposed to encapsulate nanoparticles as CT and MRI contrast into microspheres [107]. For example, Wei et al. [108] developed the magnetic embolic microspheres based on SPIONs with high density, which are detectable to both CT and MR. Additionally, gallium-based liquid metal nanoparticles have been used to realize CT/MRI dual-modality imaging, and a photothermal/photodynamic sensitizing unit for active drug release, and photothermal conversion [109].

## Conclusions

Compared with traditional procedures and the use of microspheres in interventional embolization therapy in liver cancer, novel smart microspheres and nanoparticles exhibit unique advantages, including the employment of multidrug combinations to achieve synergistic treatment, the delivery of multiple drugs to limit hypoxia-induced angiogenesis, imaging capabilities for real-time and follow-up feedback, and potential biodegradability over time. Given these advantageous characteristics, these novel smart microspheres and nanoparticles will likely overtake traditional methods in future applications. With the rapid development of new synthetic microbeads and nanoparticles in TACE for the precise treatment of HCC, it may be possible to see TACE become much more effective with less harm to the patient in the not-so-distant future.

However, apart from a few radioembolization microspheres that are commercially and clinically available, such as QuiremSpheres [110], most of the studies on novel microspheres for interventional embolization are still in the stage of animal experiments. There are also still some limitations of nanomedicines: (1) Though most reported nanoplatforms have been indicated to possess good biocompatibility in animal models, consistency may not occur in human clinical trials. The detailed pharmacokinetic, pharmacodynamic analysis, drug biodistribution, and long-term assessment of toxicity in vivo should be taken into consideration in the period of treatment; (2) Realistic factors such as production cost and the production process of nanomedicines need to be considered. Large-scale manufacturing and the maintenance of batch-to-batch quality and reproducibility are required. The development of smart microbeads for TACE is still in the early stages and more preclinical studies need to be carried out in order to progress to clinical trials.

## Data Availability

Not applicable, please refer to the original references.
